# Supplementary material for: Strong metal-support interactions induced by an ultrafast laser
Source: Nat Commun. 2021 Nov 18;12:6665. doi: 10.1038/s41467-021-27000-5 (PMC8602264; doi:10.1038/s41467-021-27000-5)
Supplement: Supplementary file 1 — Supplementary Information [file 41467_2021_27000_MOESM1_ESM.pdf]

Supplementary Information

Strong metal-support interactions induced by an ultrafast laser

Jian Zhang[1, †], Dezhi Zhu[2, †], Jianfeng Yan[2,*], Chang-An Wang[1,*]

[1]State Key Laboratory of New Ceramics and Fine Processing, School of Materials Science and Engineering, Tsinghua University, Beijing 100084, China. [2]State Key Laboratory of Tribology, Department of Mechanical Engineering, Tsinghua University, Beijing 100084, China. [†]These authors contributed equally: Jian Zhang, Dezhi Zhu. *email: yanjianfeng@tsinghua.edu.cn; wangca@tsinghua.edu.cn.

Supplementary Methods

Preparation of ceria NR

All the chemical reagent was analytically pure and without further refinement before use. The ceria NR synthesized by hydrothermal method according to the previous report. Typically, 14.4 g NaOH and 2.092 g NaCl dissolved in 50 ml deionized water to obtained solution A, 1.116 g $CeCl_3 \cdot 7H_2O$ dissolved in 10 ml deionized water to obtained solution B. Then, solution B was added to solution A drop by drop under vigorous stirring. After 30 min stirred, the homogeneous slurry transferred to stainless steel autoclave and treated at 180°C for 24 h. Finally, the ceria NR can be received after suction filtration, wash and dried.

Preparation of laser-Pt/CeO_2 NR

0.2 g Ceria NR dispersed in 180 ml deionized water under ultra-sonication to obtain homogenous slurry. Then 20 ml H_2PtCl_4 solution was added into the slurry under stirring at room temperature for 30 min. Afterwards, 6 ml newly prepared $NaBH_4$ solution (0.05 mol/ L) was dropped into the slurry under rapid stirring (800 r/min). Pt/CeO_2 NR was obtained by stirring for 2 h. The product was washed with plenty of water and dried in an oven at 60°C for 2 h, then calcined at 400°C under argon atmosphere at 400°C for 2 h to obtain fresh-Pt/CeO_2 NR. Femtosecond laser with a pulse duration of 35 fs was emitted by an amplified Ti-Sapphire system, which has a central wavelength of 800 nm and a repetition rate of 1 kHz. For the formation of laser-irradiated Pt/CeO_2, 50 mg Pt/CeO_2 powders and 2 mL deionized water were mixed into quartz cuvettes, and irradiated at femtosecond laser pulse. The exposure time was controlled at 60 min using a shutter connected to a computer, and the laser power was fixed using an attenuator at 250 mw.

Preparation of laser-Pt/TiO_2

0.2 g TiO_2 NPs (anatase) were dispersed in 180 ml deionized water under ultra-sonication to obtain a homogenous slurry. Then 4 ml H_2PtCl_4 (5 mmol/l) solution was added into the slurry under stirring at room temperature for 30 min. Afterward, 4 ml newly prepared $NaBH_4$ solution (0.01 mol/L) was dropped into the slurry under rapid stirring (800 r/min) for 1h. After separation and drying, the resulting Pt/TiO_2 catalyst was calcined in a glass tube furnace under argon atmosphere at 400°C for 2 h to obtain fresh-Pt/TiO_2. 50 mg Pt/TiO_2 powders and 2 mL deionized water were mixed into quartz cuvettes and irradiated at femtosecond laser pulse under rapid stirring for 1 h (300 r/min).

Preparation of laser-Pd/TiO_2

First, a solution mixing 21 mL of deionized water, 15 mL of H_2PdCl_4, 14 mL of absolute ethanol and 66.7 mg of PVP was refluxed in a 100 mL flask at 90 °C for 3 h to obtain a dark brown PVP-protected Pd homogeneous colloid. Subsequently, 200 mg of TiO_2 support (P25) was ultrasonically dispersed in Pd colloidal solution, stirred at room temperature for 6 h and dried in an oven at 100°C to obtain a gray powder. Then, the gray powder was calcined at 400°C under air atmosphere for 30 min to obtain fresh-Pd/TiO_2. Finally, 50 mg Pd/TiO_2 powders and 2 mL isopropanol were mixed into quartz cuvettes and irradiated at femtosecond laser pulse under rapid stirring for 1 h (300 r/min).

Preparation of laser-Au/TiO_2

Au/TiO_2 was fabricated by the deposition-precipitation method. Typically, an aqueous solution (60 mL) containing 0.04 g $HAuCl_4.3H_2O$ was adjusted to a pH of 10.0 with 1 M NaOH. After heating at 70 °C, 1 g TiO_2 (Degussa P25) was added, magnetically stirred for 2 h and then centrifuged for separation followed by washing with plenty of deionized water. The product was dried in an oven at 60°C and then calcined in air at 250°C for 2 h. Finally, 50 mg Au/TiO_2 powders and 2 mL isopropanol were mixed into quartz cuvettes and irradiated at femtosecond laser pulse under rapid stirring for 1 h (300 r/min).

Preparation of laser-Pt/Al_2O_3

Typically, 0.2 g of γ-Al_2O_3 with a diameter of about 20 nm was ultrasonically dispersed in 100 ml of deionized water, and then 4 ml of H_2PtCl_4 (5 mmol/L) was added and stirred at room temperature for 30 min to obtain a homogeneous colloid. Afterward, 4 ml newly prepared $NaBH_4$ solution (0.01 mol/L) was dropped into the slurry under rapid stirring (800 r/min) for 1 h. After separation and drying, the resulting Pt/γ-Al_2O_3 catalyst was calcined in a glass tube furnace under argon atmosphere at 400°C for 2 h to obtain fresh-Pt/γ-Al_2O_3. Finally, 50 mg Pt/γ-Al_2O_3 powders and 2 mL water were mixed into quartz cuvettes and irradiated at femtosecond laser pulse under rapid stirring for 1h (300 r/min).

Preparation of laser-Au/MgO

Typically, an aqueous solution (40 mL) containing 45 mg $HAuCl_4.3H_2O$ was adjusted to a pH of 9.0 with 1 M NaOH. After heating at 70 °C, 1 g MgO was added (the pH was kept at ~9), magnetically stirred for 2 h and then centrifuged for separation followed by washing with plenty of deionized water. The product was dried in an oven at 60°C and then calcined in air at 400°C for 2 h. Finally, 50 mg

Au/MgO powders and 2 mL water were mixed into quartz cuvettes and irradiated at femtosecond laser pulse under rapid stirring for 1h (300 r/min).

Preparation of laser-Pt/SiO_2

Typically, 0.2 g of SiO_2 with a diameter of about 20 nm was ultrasonically dispersed in 100 ml of deionized water, and then 4 ml of H_2PtCl_4 (5 mmol/l) was added and stirred at room temperature for 30 min to obtain a homogeneous colloid. Afterward, 4 ml newly prepared $NaBH_4$ solution (0.01 mol/L) was dropped into the slurry under rapid stirring (800 r/min) for 1h. After separation and drying, the resulting Pt/SiO_2 catalyst was calcined in air at 400°C for 2 h to obtain fresh- Pt/SiO_2. Finally, 50 mg Pt/SiO_2 powders and 2 mL water were mixed into quartz cuvettes and irradiated at femtosecond laser pulse under rapid stirring for 1h (300 r/min).

Finite-Difference Time-Domain Simulations (FDTD)

FDTD simulations (Lumerical Solutions) were conducted to calculate the near-field and far-field properties of the supported metal NPs. Geometric parameters of the Pt and CeO_2 NPs were extracted from TEM images. The Pt NPs were modeled as spheres of 10 nm in diameter. Dielectric permittivity tabulated by Werner[1] were used. Calculations were conducted for NPs in water. Calculations of extinction cross-sections were performed for an isolated nanostructure, which was excited at linearly polarized light. The total extinction cross-section was the average of longitudinal and transverse excitations. The distributions of enhanced electric fields were calculated for a parallel polarization. A uniform mesh-grid of 0.5 nm was used to ensure small structural details and good numerical convergence.

A finite element method is used to obtain the electromagnetic field and energy deposition of the supported metal NPs. The electromagnetic analysis is performed by solving Maxwell's equations:

$$\nabla \times \mathbf{H} = \mathbf{J} + \frac{\partial \mathbf{D}}{\partial t} \quad (1)$$

$$\nabla \times \mathbf{E} = -\frac{\partial \mathbf{B}}{\partial t} \quad (2)$$

$$\nabla \cdot \mathbf{D} = \rho \quad (3)$$

$$\nabla \cdot \mathbf{B} = 0 \quad (4)$$

where **E** is the electric field intensity; **D** is the electric displacement; **H** is the magnetic field intensity;

B is the magnetic flux density; **J** is the current density, and ρ is the electric charge density.

To obtain a closed system, the constitutive relations of the material properties must be included. They are given as follows:

$$\mathbf{D} = \varepsilon \mathbf{E} \tag{5}$$

$$\mathbf{B} = \mu \mathbf{H} \tag{6}$$

$$\mathbf{J} = \sigma \mathbf{E} \tag{7}$$

where ε and μ are the permittivity and permeability of the material, respectively; σ is the conductivity of the material. By solving the Maxwell equations, the local electric field is obtained.

Supplementary Figures

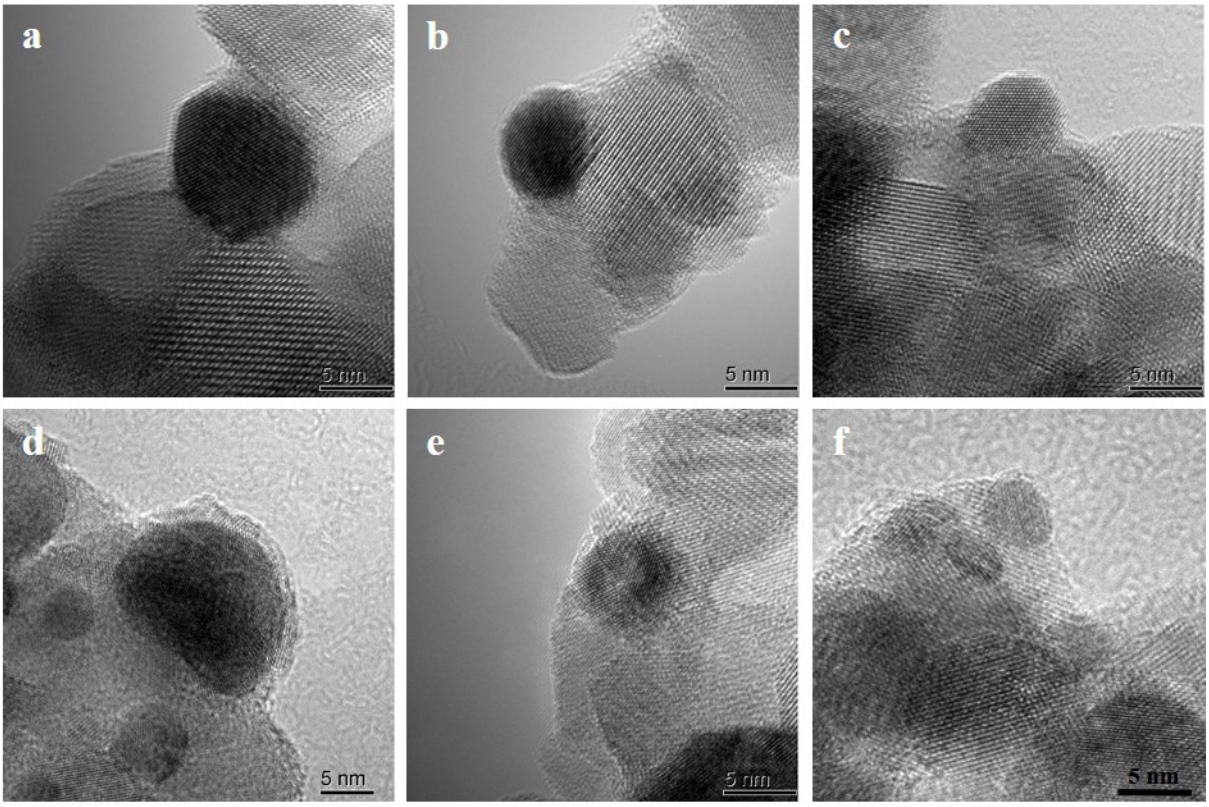

Supplementary Fig 1. **a-c** TEM images of fresh-Pt/CeO_2. **d-f** TEM images of laser-irradiated Pt/CeO_2.

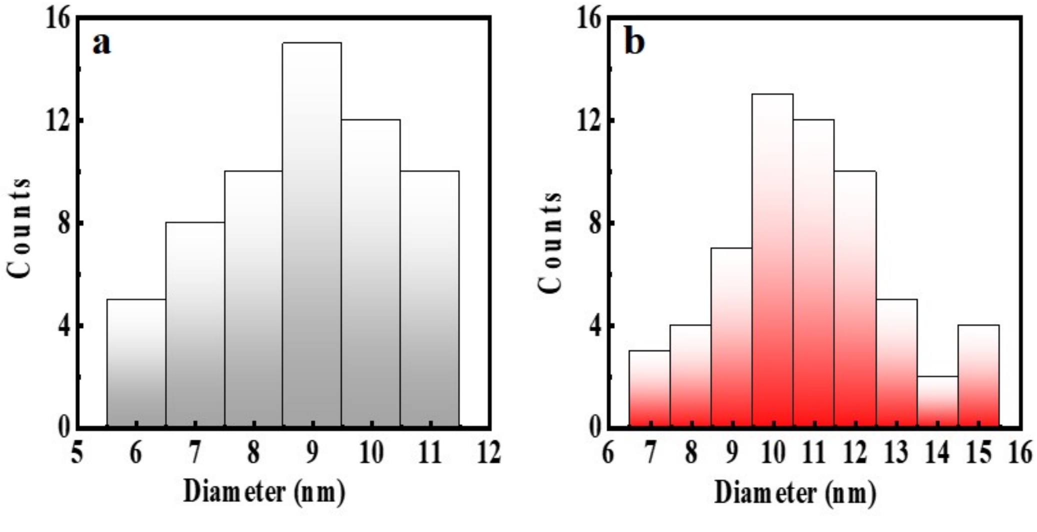

Supplementary Fig 2. The size distributions of Pt NPs **a** before and **b** after laser irradiation deduced from statistics of TEM images.

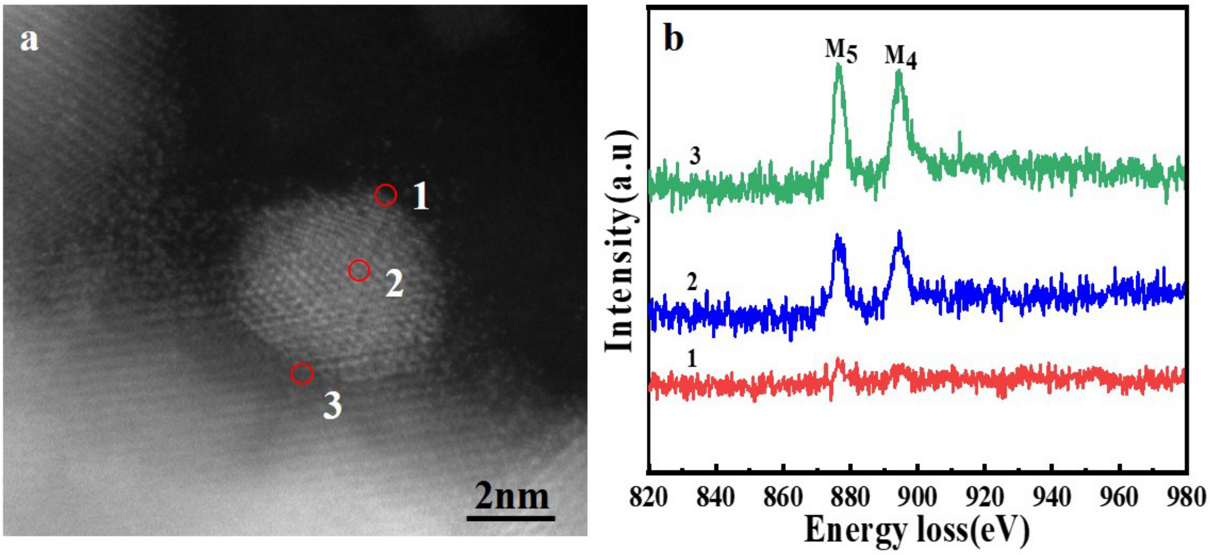

Supplementary Fig 3. a High-angle annular dark-field scanning transmission electron microscopy (HAADF-STEM) image of laser-irradiated Pt/CeO_2. **b** EELS spectrum of laser-irradiated Pt/CeO_2.

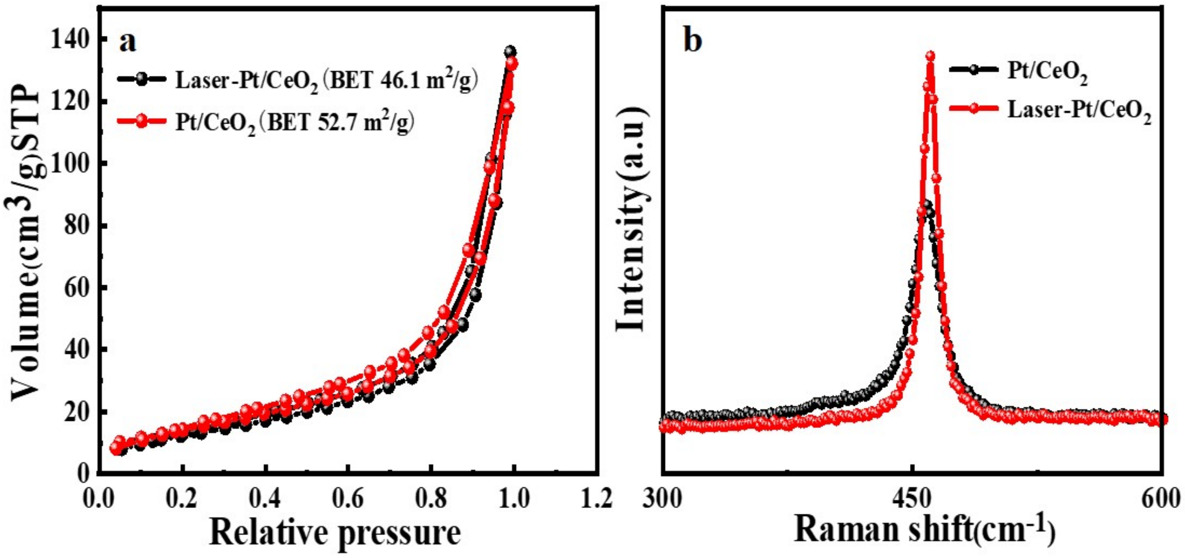

Supplementary Fig 4. a BET surface area of Pt/CeO_2 and laser-irradiated Pt/CeO_2. **b** Raman spectra of Pt/CeO_2 and laser-irradiated Pt/CeO_2.

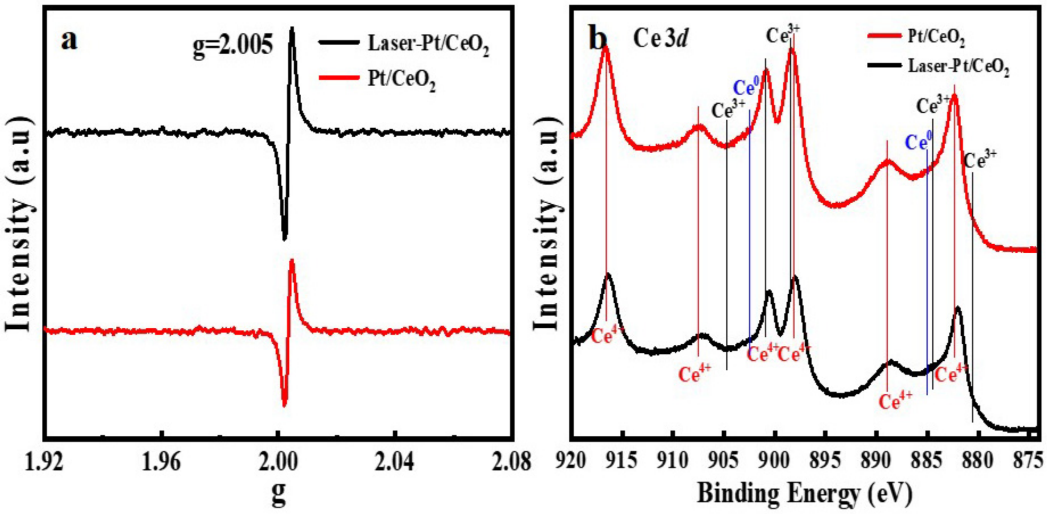

Supplementary Fig 5. a EPR spectra (at 77K) of Pt/CeO_2 and laser-irradiated Pt/CeO_2. **b** XPS spectra of Pt/CeO_2 and laser-irradiated Pt/CeO_2.

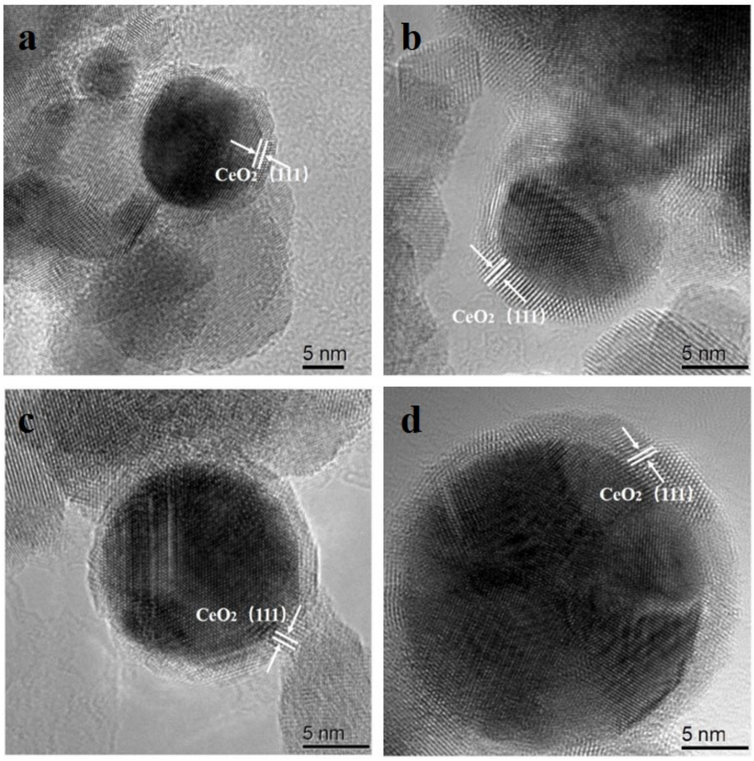

Supplementary Fig 6. a-d HRTEM images of laser-irradiated Pt/CeO_2

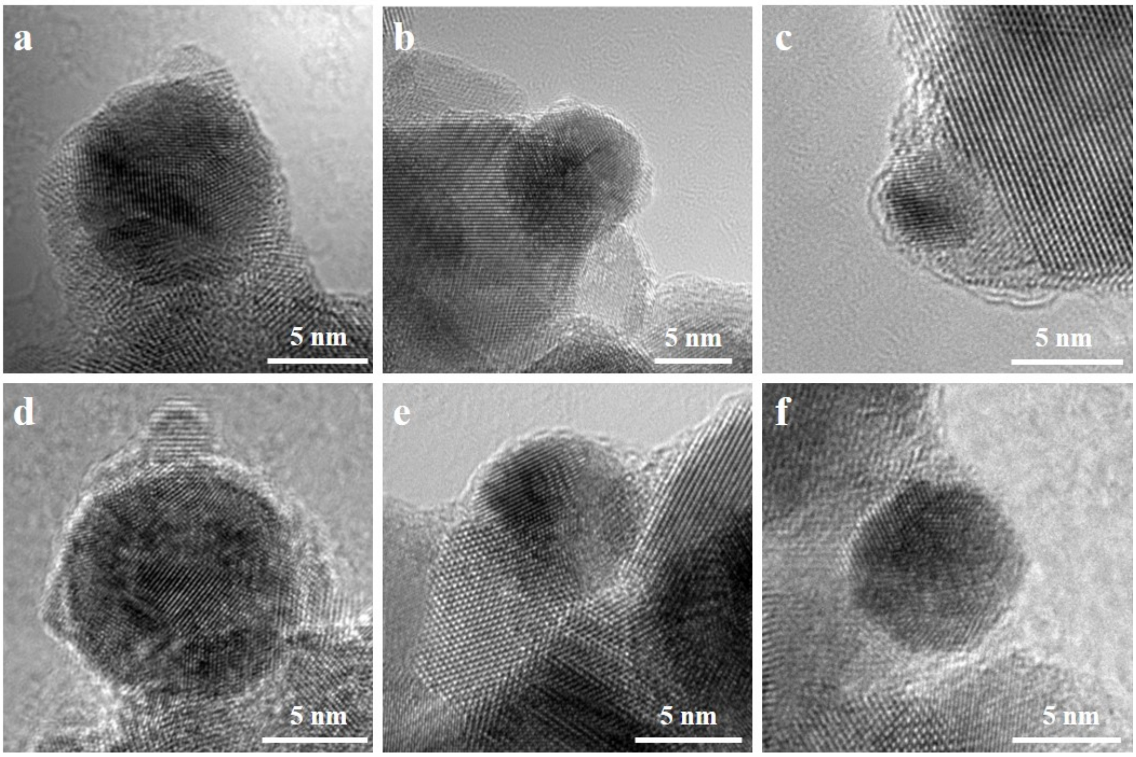

Supplementary Fig 7. a-f TEM images of laser-irradiated Pt/CeO_2

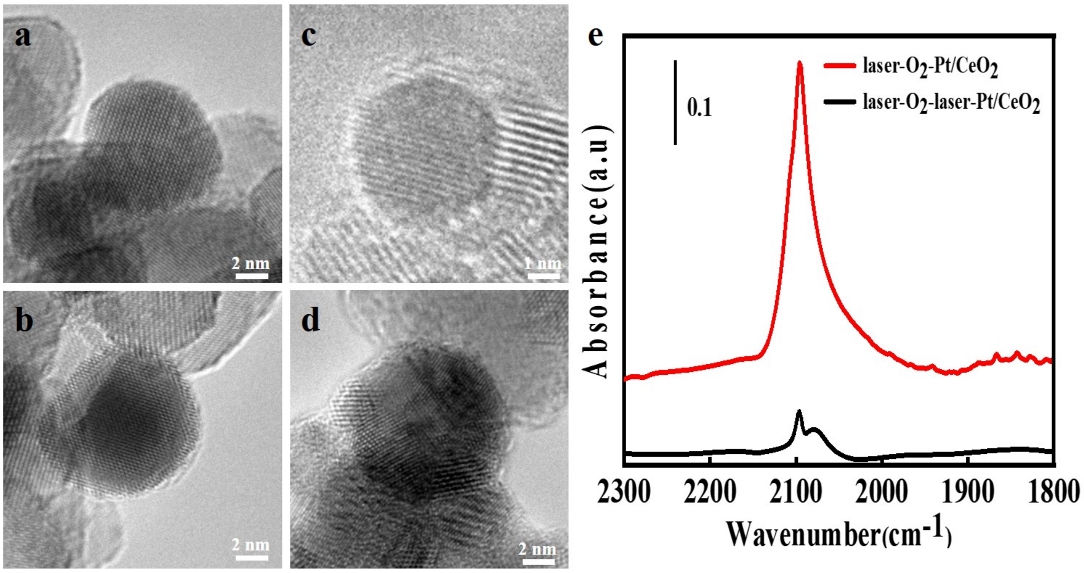

Supplementary Fig 8. TEM images of **a, b** laser-O_2-Pt/CeO_2; **c, d** laser-O_2-laser-Pt/CeO_2. **e** CO-DRIFT spectra of laser- O_2-Pt/CeO_2 and laser-O_2-laser-Pt/CeO_2.

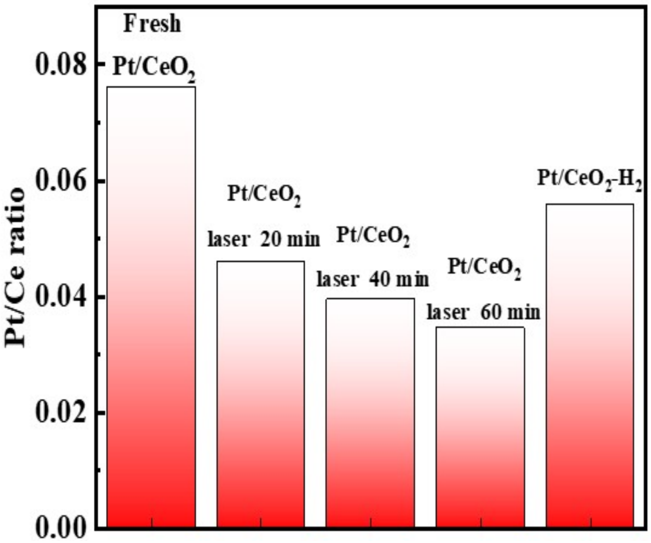

Supplementary Fig 9. XPS results of the Pt/Ce ratio on the surface of specimens (Fresh-Pt/CeO_2, Pt/CeO_2-laser-20 min, Pt/CeO_2-laser-40 min, Pt/CeO_2-laser-60 min, and Pt/CeO_2-H_2).

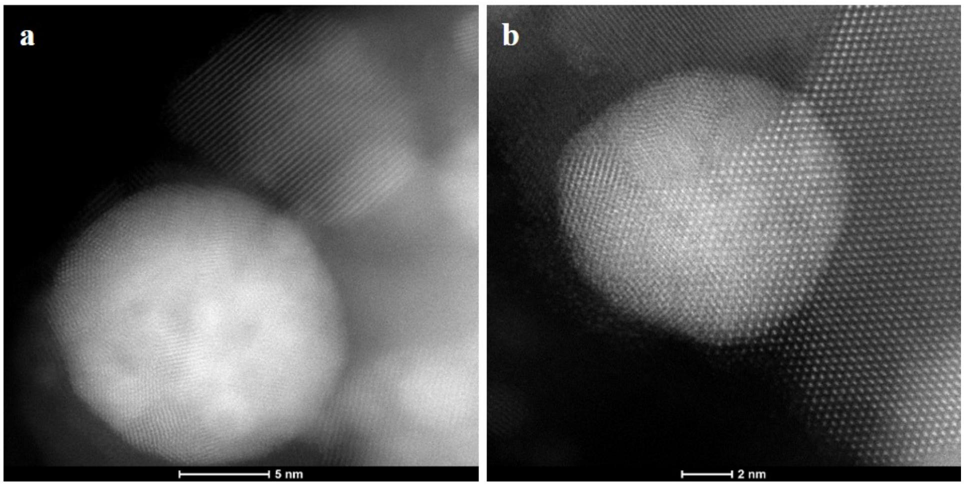

Supplementary Fig 10. a, b HAADF-STEM images of laser-irradiated Pt/CeO_2.

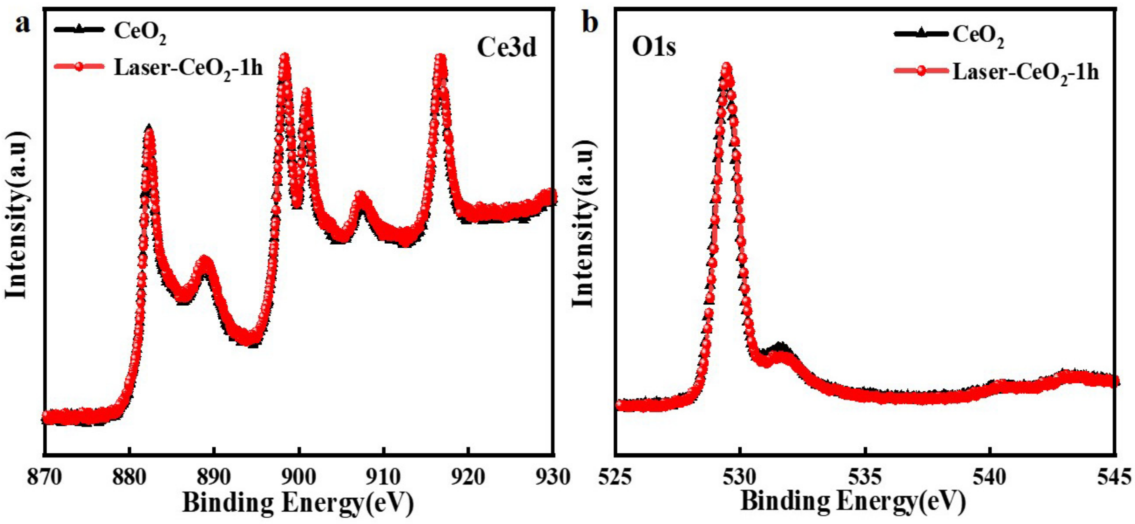

Supplementary Fig 11. a, b XPS spectra of CeO_2 and laser-irradiated CeO_2.

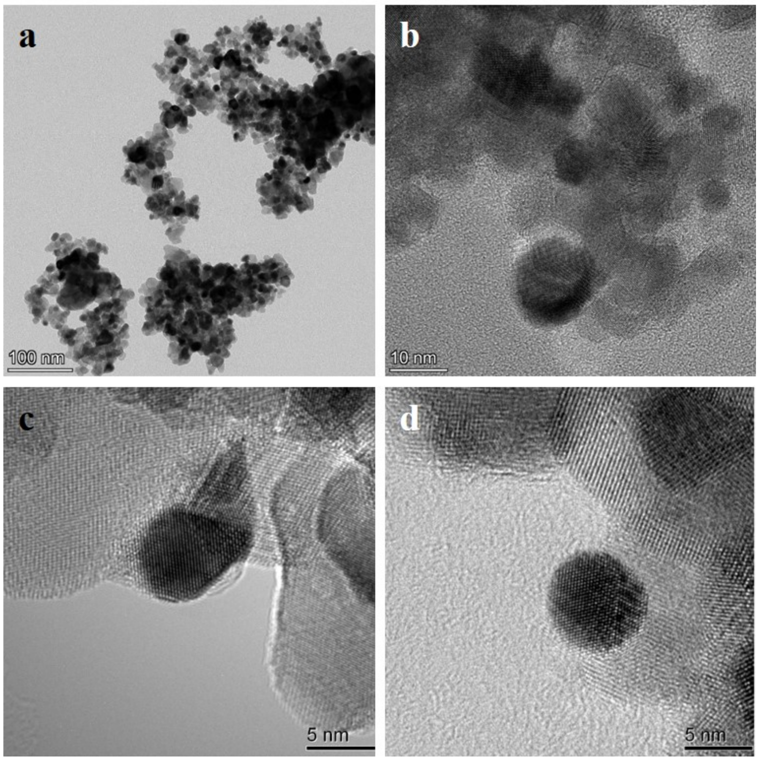

Supplementary Fig 12. a-d TEM images of laser-treated CeO_2 loaded with Pt NPs

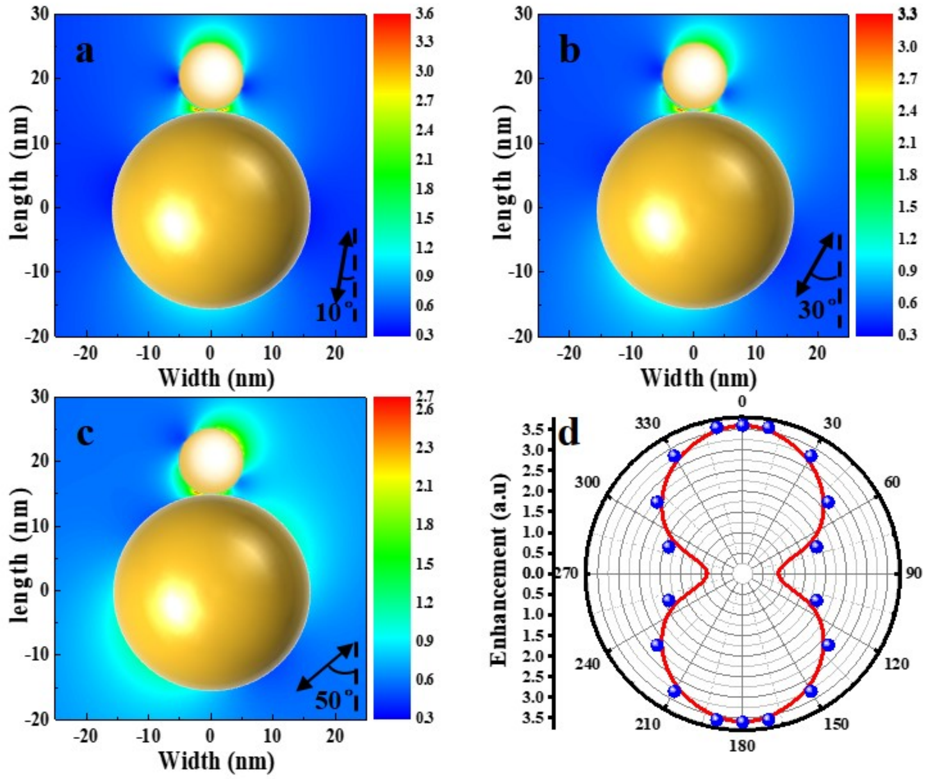

Supplementary Fig 13. Calculated electric field distribution of Pt/CeO_2 irradiated with 400-nm laser pulse at polarization angles of **a** 10°, **b** 30° and **c** 50°. **d** Simulated dependence of the enhanced electric field at the interface between Pt and CeO_2 on the polarization angles.

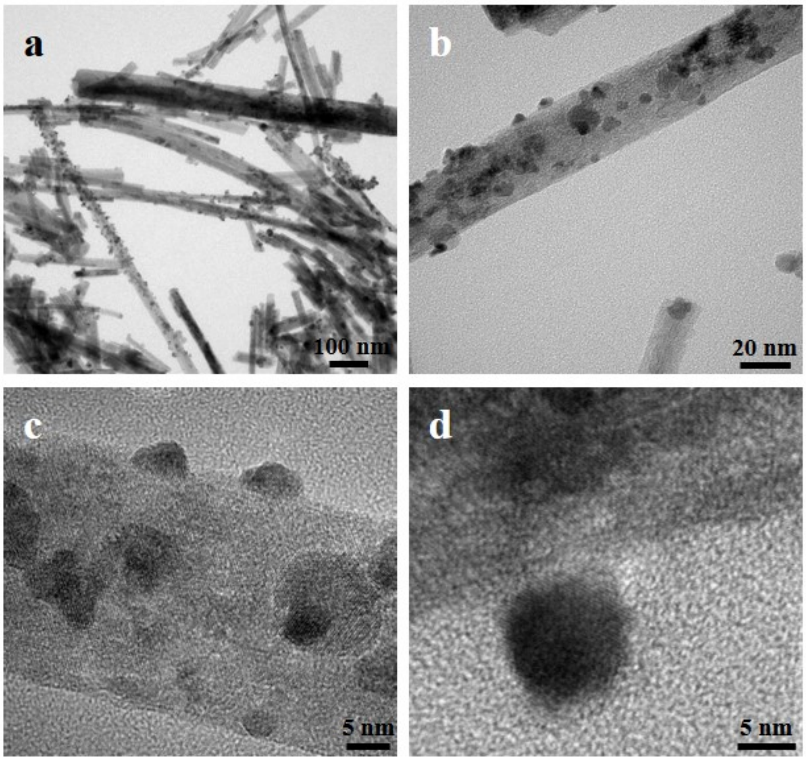

Supplementary Fig 14. a-d TEM images of as-prepared Pt/CeO_2 NRs.

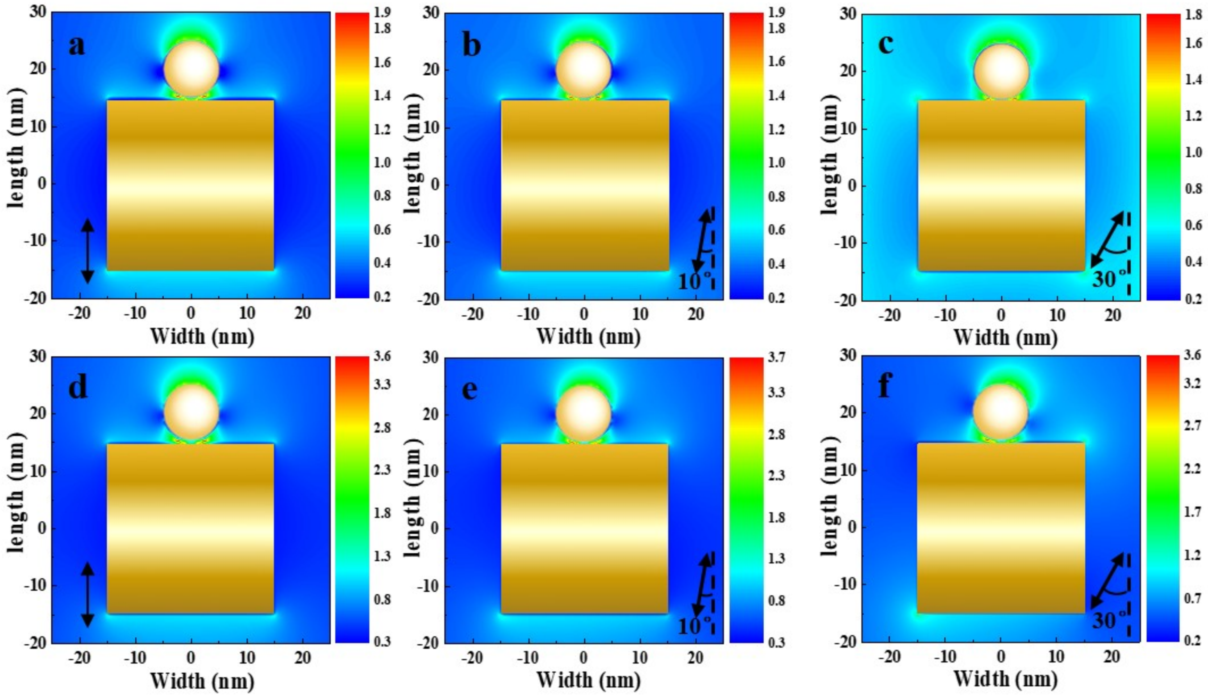

Supplementary Fig 15. Calculated electric field distribution of Pt/CeO_2 NRs irradiated with **a-c** 800-nm and **d-f** 400-nm laser pulse at polarization angles of 0°, 10° and 30°.

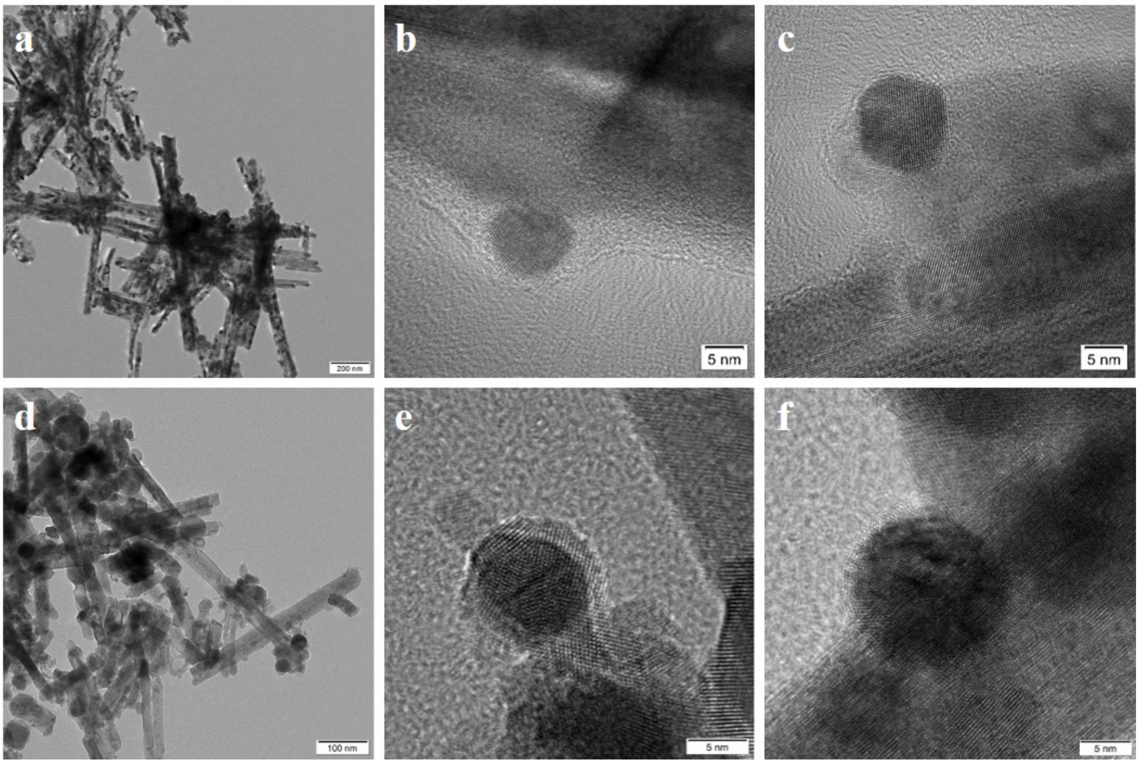

Supplementary Fig 16. a-f TEM images of laser-irradiated Pt/CeO_2 NRs.

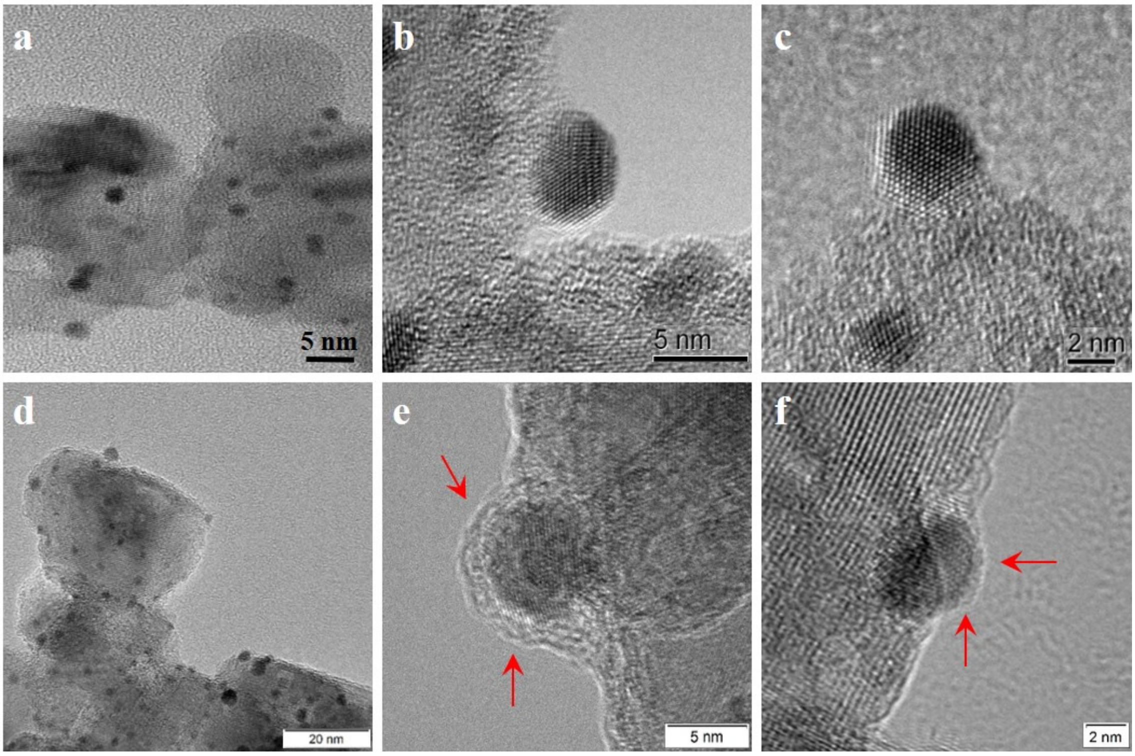

Supplementary Fig 17. a-c TEM images of fresh-Pt/TiO_2. **d-f** TEM images of laser-irradiated Pt/TiO_2.

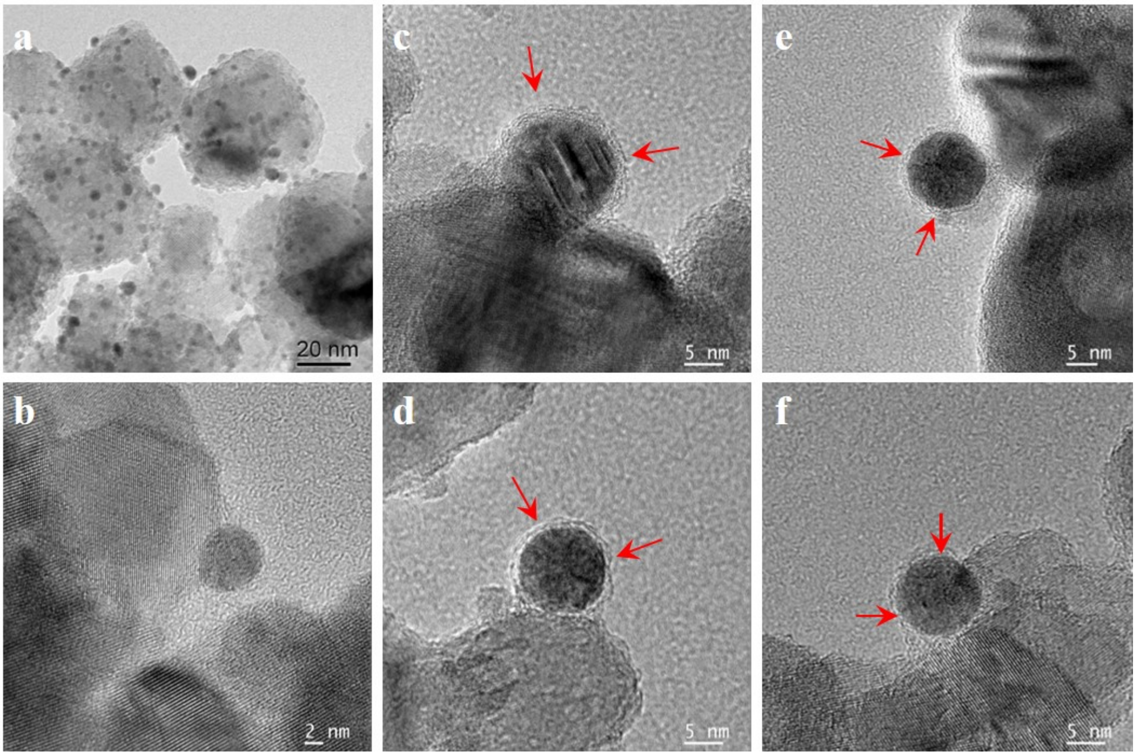

Supplementary Fig 18. a, b TEM images of fresh- Pd/TiO_2. **c-f** TEM images of laser-irradiated Pd/TiO_2.

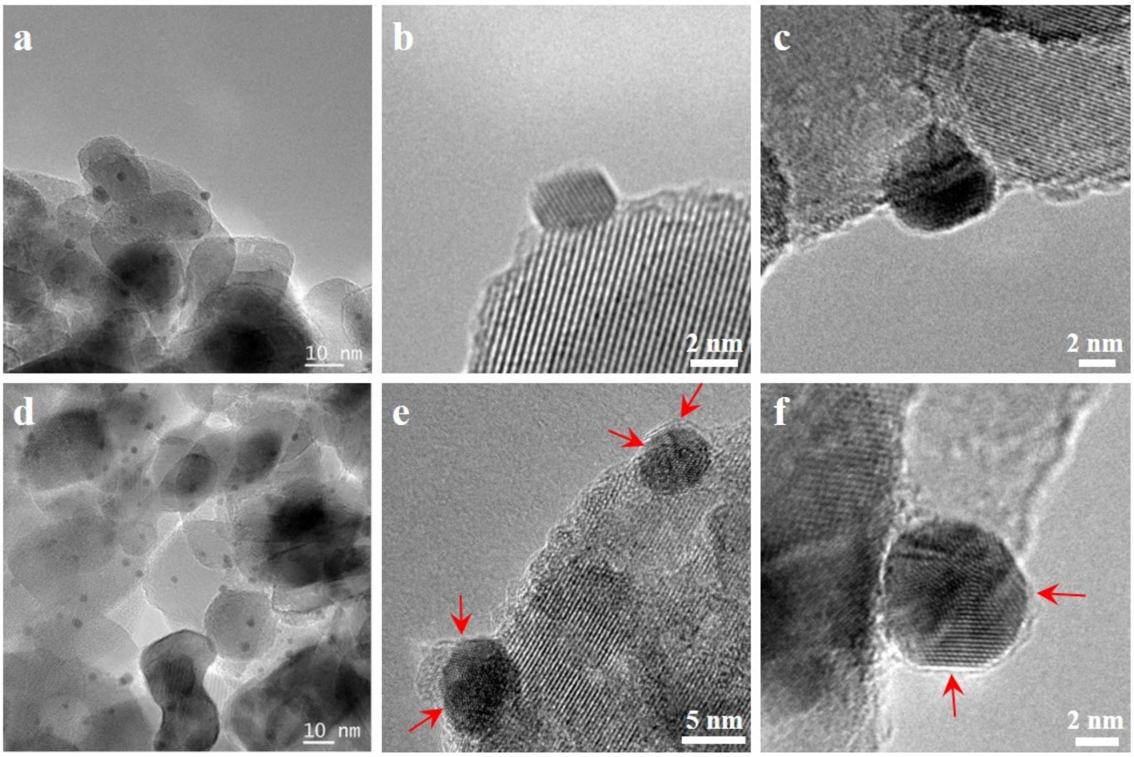

Supplementary Fig 19. a-c TEM images of fresh- Au/TiO_2. **d-f** TEM images of laser-irradiated

Au/TiO_2.

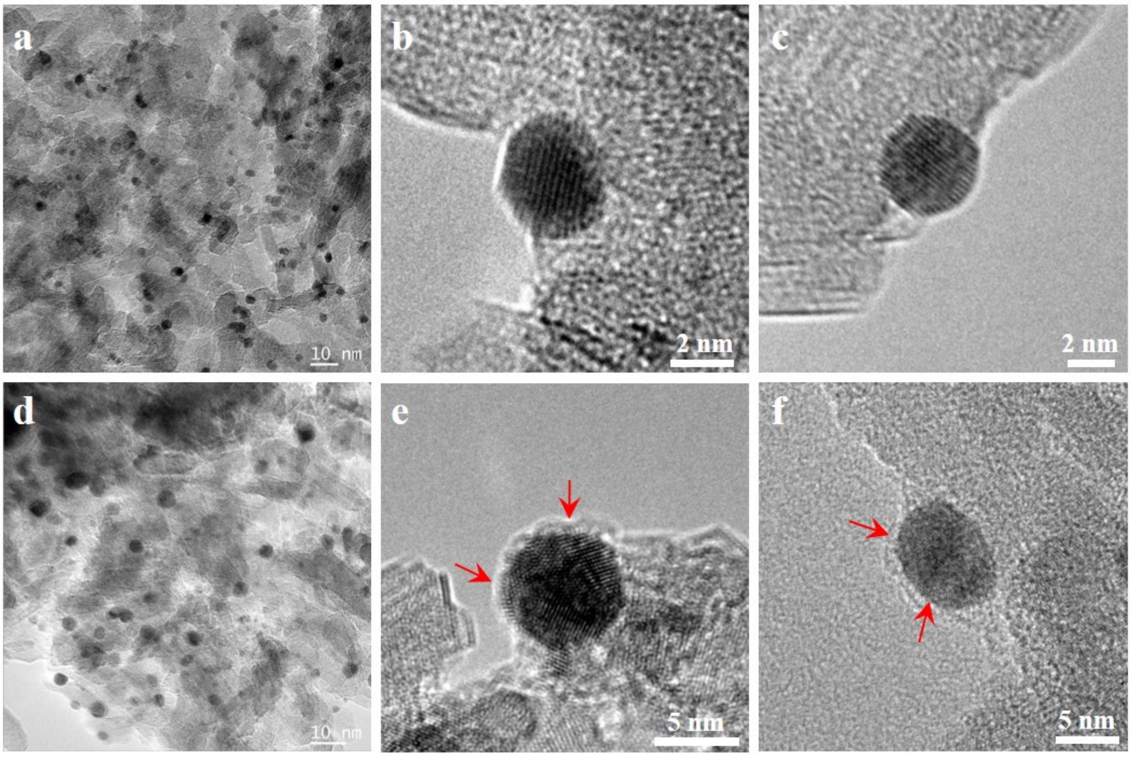

Supplementary Fig 20. a-c TEM images of fresh- Pt/Al_2O_3. **d-f** TEM images of laser-irradiated Pt/Al_2O_3.

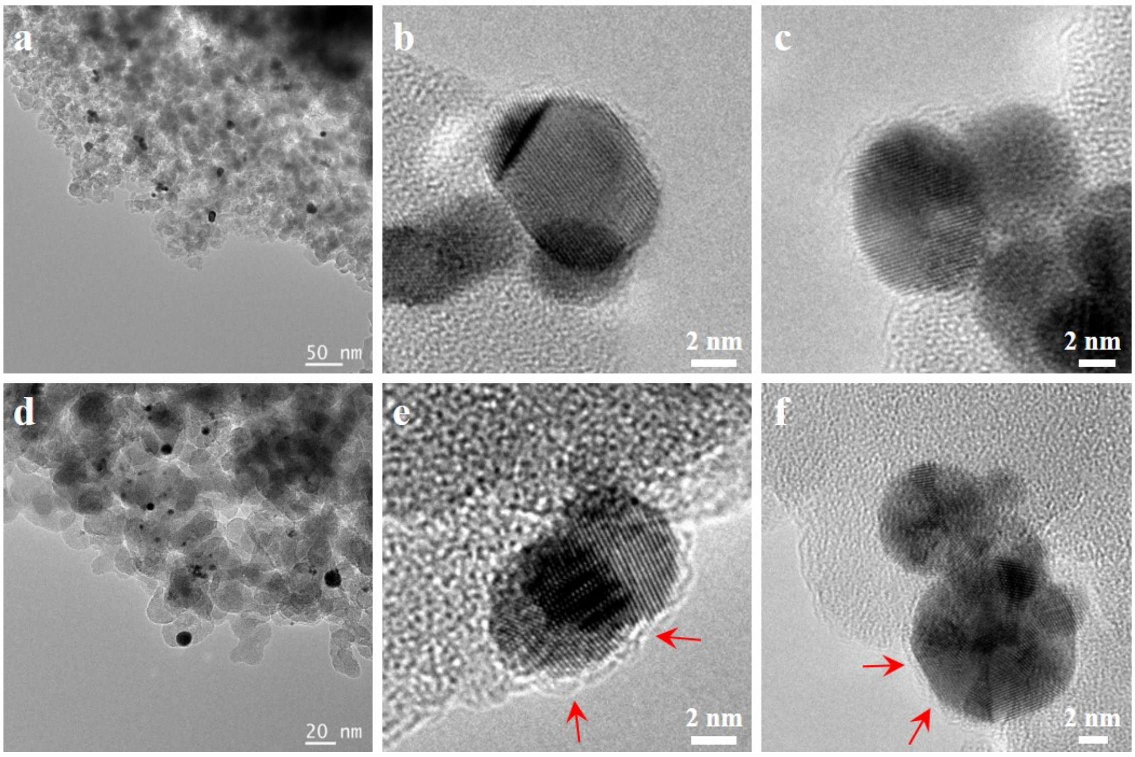

Supplementary Fig 21. a-c TEM images of fresh- Pt/SiO_2. **d-f** TEM images of laser-irradiated Pt/SiO_2.

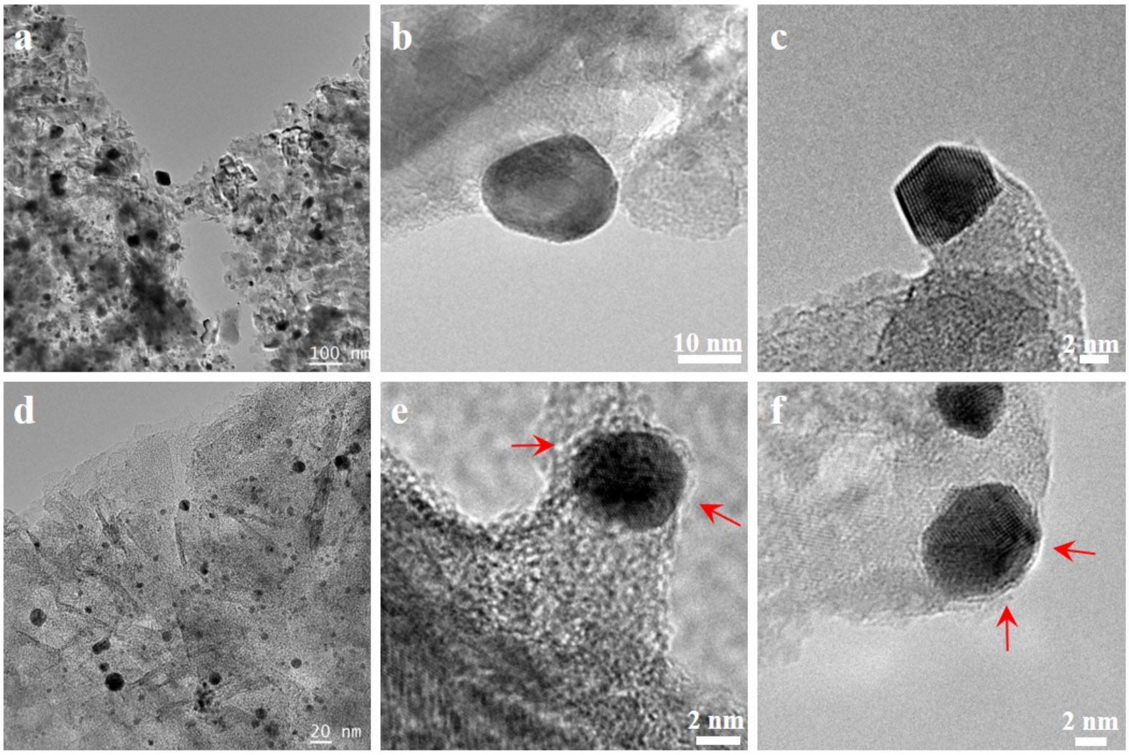

Supplementary Fig 22. a-c TEM images of fresh- Au/MgO. **d-f** TEM images of laser-irradiated Au/MgO.

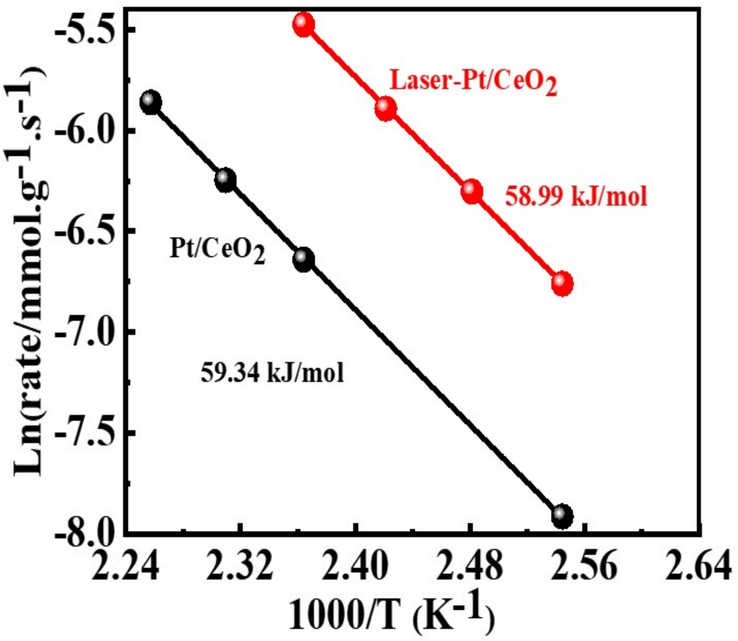

Supplementary Fig 23. Arrhenius plots of CO conversion rates.

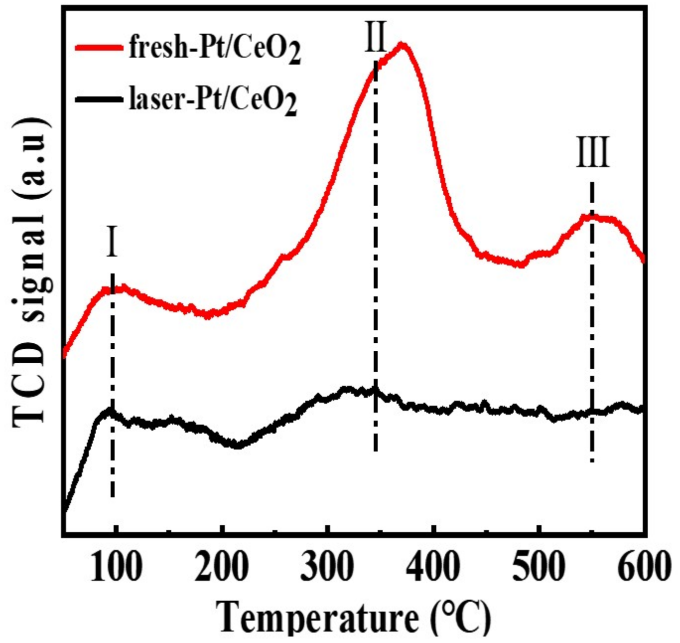

Supplementary Fig 24. H_2-TPD spectra of fresh Pt/CeO_2 and laser-irradiated Pt/CeO_2

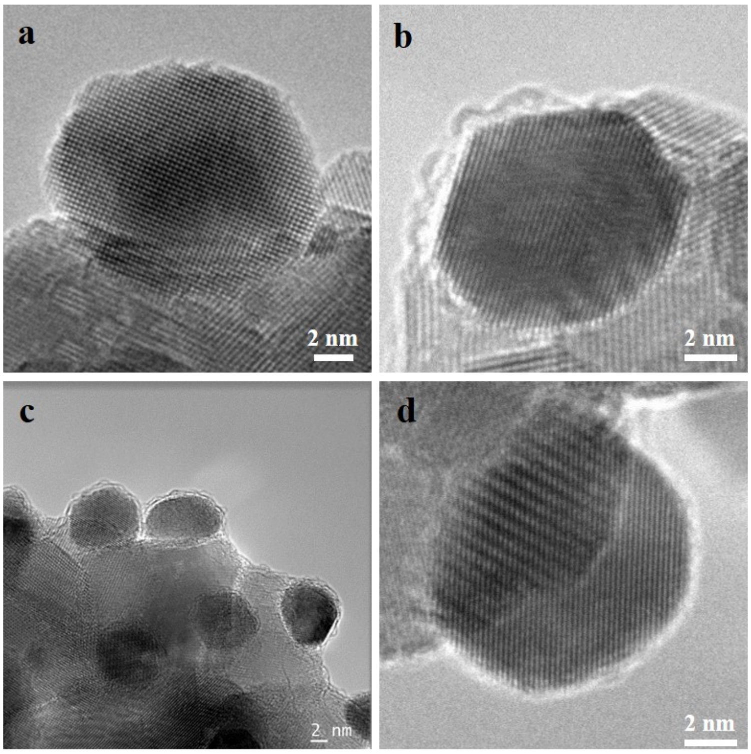

Supplementary Fig 25. TEM images of **a** fresh-Pt/CeO_2; **b, c** Pt/CeO_2- $H_2$700ºC; **d** Pt/CeO_2- $H_2$700ºC -$O_2$600ºC

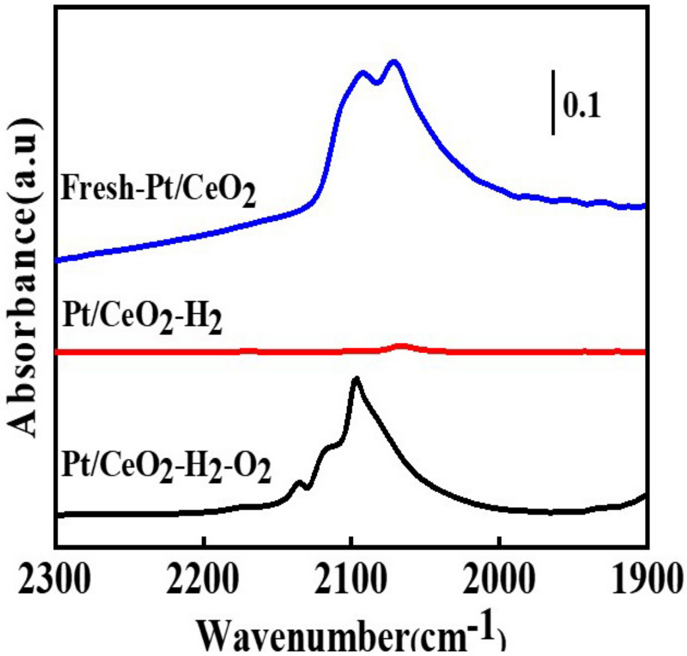

Supplementary Fig 26. In situ CO-DRIFT of fresh Pt/CeO_2 and H_2-Pt/CeO_2

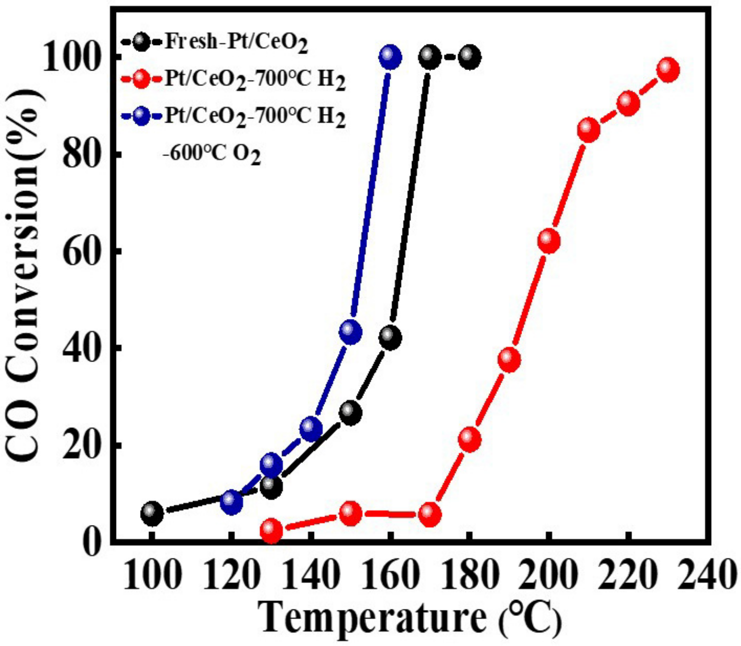

Supplementary Fig 27. CO oxidation curves of fresh-Pt/CeO_2, H_2-Pt/CeO_2 and H_2-Pt/CeO_2-O_2 catalyst.

Supplementary Tables

Supplementary Table 1. Comparison of the SMSI construction between the previous procedures[2-11] and the pathway used in this work.

	Material	Support properties	Method	Reference
1	Au/TiO_2	Reductive	H_2 treatment	*Sci. Adv.* 2017, 3, 1700231; *Nat. Commun*. 2020, 11, 5811
2	Au/TiO_2	Reductive	Melamine treatment	*Nat. Commun.* 2019, 10, 5790
3	Au/TiO_2	Reductive	Wet-chemistry	*J. Am. Chem. Soc.* 2019, 141, 2975
4	Au/TiO_2	Reductive	Sacrificial coating	*J. Am. Chem. Soc.* 2016, 138, 16130.
5	Au/TiO_2	Reductive	Thermal annealing	*Angew. Chem. Int. Ed.* 2017, 56, 4494
6	Pt/TiO_2	Reductive	H_2 treatment	*Nat. Commun*. 2020, 11, 3220
7	Au/MgO	Irreductive	CO_2 treatment	*Nat. Catal.* 2021, 4, 418
8	Au/SiO_2	Irreductive	Deposition-precipitation	*Nat. Commun*. 2020, 11, 558
9	Au/ hydroxyapatite	Irreductive	H_2 treatment	*J. Am. Chem. Soc.* 2016, 138, 56
10	Pt/CeO_2, Pt/TiO_2, Pd/TiO_2, Au/TiO_2, Pt/Al_2O_3, Pt/SiO_2, and Au/MgO	Reductive /irreductive	Ultrafast laser treatment	**This work**

Supplementary References

1. Werner, W. S., Glantschnig, K. & Ambrosch-Draxl, C. Optical constants and inelastic electron-scattering data for 17 elemental metals. *J Phys Chem Ref Data* **38**, 1013-1092 (2009).
2. Tang, H. *et al.* Classical strong metal–support interactions between gold nanoparticles and titanium dioxide. *Science advances* **3**, e1700231 (2017).
3. Du, X. *et al.* Size-dependent strong metal-support interaction in TiO 2 supported Au nanocatalysts. *Nat Commun* **11**, 1-8 (2020).
4. Liu, S. *et al.* Ultrastable Au nanoparticles on titania through an encapsulation strategy under oxidative atmosphere. *Nat Commun* **10**, 5790 (2019).
5. Zhang, J. *et al.* Wet-Chemistry Strong Metal-Support Interactions in Titania-Supported Au Catalysts. *J Am Chem Soc* **141**, 2975-2983 (2019).
6. Zhan, W. *et al.* A sacrificial coating strategy toward enhancement of metal–support interaction for ultrastable Au nanocatalysts. *J Am Chem Soc* **138**, 16130-16139 (2016).
7. Zhan, W. *et al.* Surfactant-Assisted Stabilization of Au Colloids on Solids for Heterogeneous Catalysis. *Angewandte Chemie International Edition* **56**, 4494-4498 (2017).
8. Beck, A. *et al.* The dynamics of overlayer formation on catalyst nanoparticles and strong metal-support interaction. *Nat Commun* **11**, 3220 (2020).
9. Wang, H. *et al.* Strong metal–support interactions on gold nanoparticle catalysts achieved through Le Chatelier's principle. *Nat Catal* **4**, 418-424 (2021).
10. Zhang, Y. *et al.* Boosting the catalysis of gold by O2 activation at Au-SiO2 interface. *Nat Commun* **11**, 558 (2020).
11. Tang, H. *et al.* Strong Metal-Support Interactions between Gold Nanoparticles and Nonoxides. *J Am Chem Soc* **138**, 56-59 (2016).
